# Ultra-broadband terahertz absorption by exciting the orthogonal diffraction in dumbbell-shaped gratings

**DOI:** 10.1038/srep08901

**Published:** 2015-03-10

**Authors:** XiaoFei Zang, Cheng Shi, Lin Chen, Bin Cai, YiMing Zhu, SongLin Zhuang

**Affiliations:** 1 Shanghai Key Lab of Modern Optical System and Engineering Research Center of Optical Instrument and System, Ministry of Education, University of Shanghai for Science and Technology, No. 516 JunGong Road, Shanghai 200093, People's Republic of China

## Abstract

Metamaterials, artificial electromagnetic media consisting of periodical subwavelength metal-based micro-structures, were widely suggested for the absorption of terahertz (THz) waves. However, they have been suffered from the absorption of THz waves just in the single-frequency owing to its resonance features. Here, in this paper, we propose a simple periodical structure, composed of two 90 degree crossed dumbbell-shaped doped-silicon grating arrays, to demonstrate broadband THz wave absorption. Our theoretical and experimental results illustrate that THz waves can be efficiently absorbed more than 95% ranging from 0.92 THz to 2.4 THz. Such an ultra-wideband polarization-independent THz absorber is realized mainly based on the mechanisms of the anti-reflection effect together with the [±1, 0]-order and [0, ±1]-order grating diffractions. The application of our investigation can be extend to THz couplers, filters, imaging, and so on.

Electromagnetic metamaterials, made of densely artificial arranged subwavelength resonance cells, have produced exotic effects such as invisible cloaking, negative refraction, Fano resonance and superlens^1^,^2^,^3^,^4^,^5^,^6^,^7^,^8^. Recently, metamaterial-based perfect absorbers have attracted a great deal of interests in worldwide due to a host of potential applications including imaging, detecting, and sensing^9^,^10^,^11^. The first single-frequency micro-wave metamaterial perfect absorber (MPA) was proposed by N. I. Landy *et al*., and it was composed of two metallic layers separated with a loss dielectric spacer to couple the electric and magnetic fields, respectively^12^. H. Tao *et al.*, also designed a ring resonator (SRR)/loss dielectric spacer/metal-wire sandwich ‘fishnet’ MPA to realize a single-frequency and polarization-dependent THz absorber with absorbance of 70%^13^. Then, polarization-independent single-frequency THz absorber was obtained based on the interference effects by using a symmetrical metal-based ERR and a mirror underneath the ERR^14^. Here, the SRR is designed to change the phase of the transmitted and the reflected waves. The loss dielectric spacer and the metal-substrate are applied to absorb the incident waves and avoid the transmission of the incident waves, respectively. At certain frequency, all of the reflected waves are destructively coherent with each other, and thus no incident wave is reflected, resulting in a single-frequency of MPA. According to the destructive coherent effects, dual-band, triple-band, and multi-band THz absorbers were demonstrated by embedding much more ERR with different sizes or shapes^15^,^16^,^17^,^18^,^19^,^20^. Broadband THz absorbers were also realized by introducing multi-layered (much more than two layers) gradually varied ERR structure^21^,^22^,^23^. In addition, many similar structured absorbers have been widely investigated in microwave and optical frequencies^24^,^25^,^26^,^27^,^28^,^29^,^30^,^31^. But, all of these multi-band and broadband THz absorbers are suffered from the difficulties of either alignment or fabrication (complex structure), which hinders their practical application.

As a practical solution, R. Kakimi *et. al*., proposed a single-layered photonic-crystal slab, which can be easily fabricated with doped silicon, to capture of THz waves^32^. The distributed Bragg reflections in this structure create photonic bandgaps, which can be utilized for wave confinement. So, when they operate the crystals at the photonic band edges (leaky-mode regime at the photonic band edges), the in-plane guide wave and the free-space wave incident from the out-of-plane direction can be strongly coupled with each other, resulting in the guide-mode resonance (in-plane resonant modes). In addition, a mirror was introduced underneath the photonic-crystal slab to induce Fabry–Pérot resonance. Therefore, a 50 GHz (with absorptivity ≥ 90%) bandwidth of THz absorber was realized by combining the guided-mode resonance and the Fabry–Pérot resonance. Recently, M. Pu *et. al*., have theoretically reported another simple structure *i.e.*, doped silicon with square-shaped grating arrays, to realize a much wider bandwidth of THz absorption in virtue of the anti-reflection effects and the first-order diffraction^33^, and the experimental demonstration was realized in our previous work^34^. However, the square-shaped grating array just utilize the [±1, 0]-order grating diffraction, which suppresses the bandwidth of the absorber. Here, in this paper, different from the above broadband absorbers based on the destructive coherent effects, our ultra-broadband THz absorber is realized by using the anti-reflection effects, especially the [±1, 0]-order and the [0, ±1]-order grating diffractions. We achieve such an ultra-broadband THz absorber by designing a single-layered periodical structure consisting of two 90 degree crossed dumbbell-shaped doped-silicon grating. In such a single-layered dumbbell-shaped doped-silicon grating, the smaller horizontal gap between the horizontal dumbbells and the bigger horizontal gap between the vertical dumbbells are designed to excite the [±1, 0]-order and the [0, ±1]-order grating diffractions, simultaneously. And, the absorption bandwidth is further enhanced in such a single-layered grating rather than exciting the second-order grating diffraction (to enhance the absorption bandwidth) in a double-layered grating, as theoretical discussed in Ref. 33. Our numerical simulations and especially the experimental testing results demonstrate a polarization-independent THz absorber with nearly 1.5 THz absorption bandwidth (with absorptivity ≥ 95%). Here, we want to emphasis that the absorption bandwidth defined in this paper is not the full width at half maximum (FWHM). As defined in Ref. 32, our absorption bandwidth means the corresponding bandwidth for absorptivity ≥ 95%. To the best of our knowledge, such an absorption bandwidth with absorptivity ≥ 95% (especially, our experimental results) is much broader than all of the previous reported THz absorbers.

## Results and Discussion

**Figure 1:**
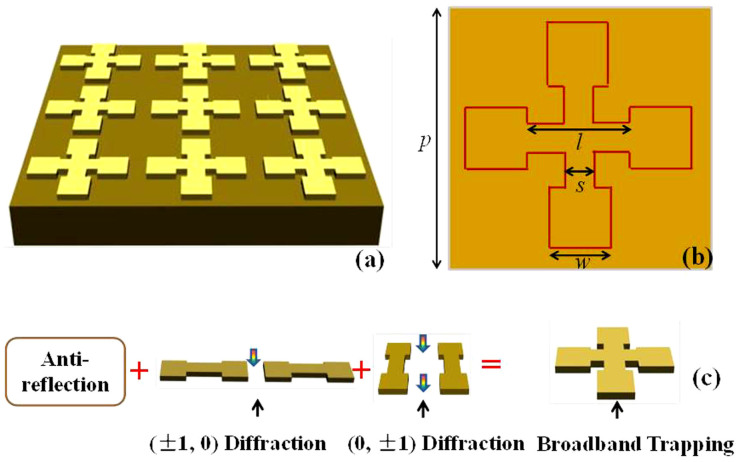
Schematics illustrating the (a) periodical structure and (b) unit cell of the THz wave absorber. (c) The fundamental principal of THz wave absorption for broadband operation.

The schematic and the fundamental principal of such an ultra-broadband THz absorber are shown in Fig. 1. Our model system has a square lattice with 90 degree rotational symmetry, for which the absorption is insensitive to the polarization of the incident THz waves (Fig. 1(a)). Each unit cell consists of two 90 degree crossed dumbbell-shaped doped-silicon strips (Fig. 1(b)). The corresponding geometric parameters are *p* = 96 μm, *l* = 27 μm, *s* = 17 μm, and *w* = 25 μm. The thickness (*h*) of each dumbbell-shaped strip (pattern) and substrate are 38 μm and 462 ± 10 μm, respectively. The permittivity of both the dumbbell-shaped doped-silicon grating and the substrate can be described by Durde model as follows:
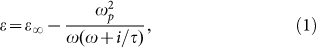
where *ε*_∞_ = 11.7, *τ* is the carrier relaxation time, and *ω_p_* is the plasmon frequency. In this paper, the resistivity of the boron-doped silicon is 0.54 Ω cm, and thus the corresponding *τ* is 0.571 ps, and *ω_p_* is 19.1 THz. The fundamental principal of our model system is shown in Fig. 1(c). In the low frequency regime, the dumbbell-shaped doped-silicon grating is equivalent to an effective medium coating on the substrate, and the absorption of THz waves is mainly attributed to the anti-reflection effect between the incident and the reflected THz waves. However, in high frequency regime, these dumbbell-shaped strip arrays can be considered as two 90 degree crossed grating (the horizontal stripe and the vertical stripe grating array). Therefore, the [±1, 0]-order and [0, ±1]-order grating diffractions are separately contributed to the absorption of THz waves. By choosing the proper structure parameters (as shown above), the anti-reflection effect and the grating diffractions can be jointed with each other, resulting in a wide-bandwidth of THz absorber. Here, we want to emphasis that such a crossed dumbbell-shaped doped-silicon grating array is designed through the parameters optimization based on numerical simulations. Although a cross-shaped doped-silicon grating can also be designed as a broadband THz absorber, the corresponding bandwidth is less than the crossed dumbbell-shaped doped-silicon grating (The numerical simulations are not shown here).

**Figure 2:**
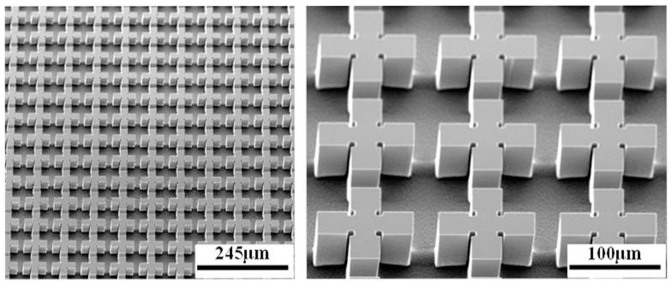
SEM of the fabricated periodical array slab.

Figure 2 shows the scanning electron microscope (SEM) images of the dumbbell-shaped doped-silicon grating. The testing sample is fabricated by using the traditional photolithography and the inductively coupled plasma (ICP) etching on a 0.54 Ω·cm *p*-type silicon wafer (with thickness of 500 ± 10 μm). Based on a serial of technical processing, the dumbbell-shaped doped-silicon grating with the thickness of 38 μm is formed on the surface of the doped-silicon (see the methods).

**Figure 3:**
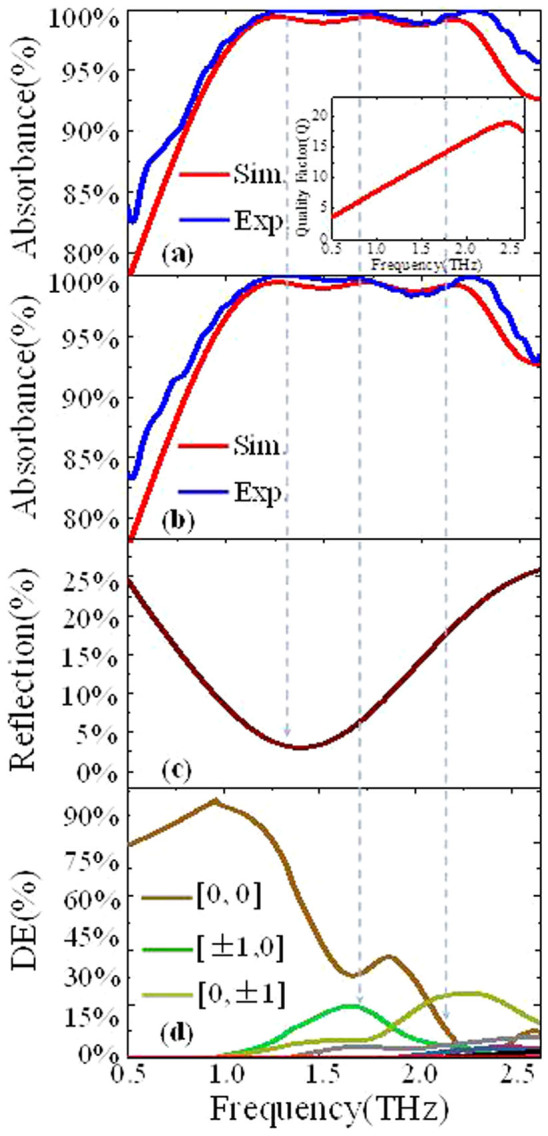
The simulated (red line) and measured (blue line) results of the polarization-independent broadband THz absorber with (a) TE incident THz wave, and (b) TM incident THz wave. (c) The calculated reflection spectrum in the case of that the periodical array on top of the substrate is equivalent as an effective medium. (d) The diffraction efficiency (DE) of different diffraction orders. The inset in (a) is the quality factor of such periodical structure.

**Figure 4:**
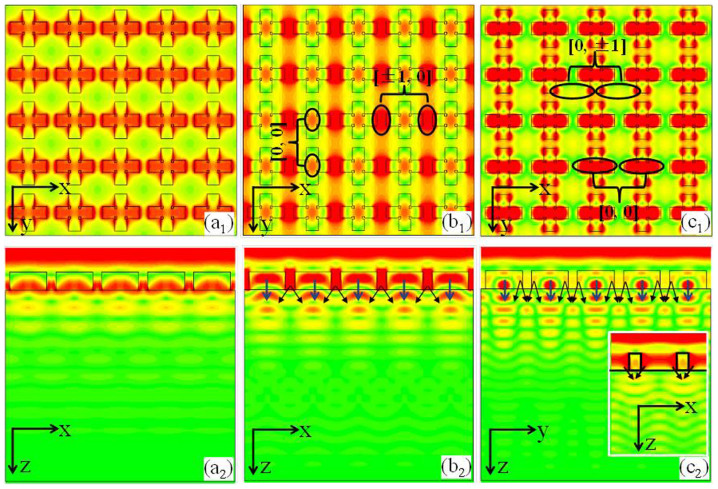
The electric field distribution at the interface between the periodical arrays and the substrate for *f* = 1.265 THz (a), *f* = 1.688 THz (b) and *f* = 2.135 THz (c), respectively. The inset in (c) is the electric field distribution at y = 38 μm.

The calculated and measured absorption spectra for the TE (transverse electric) and TM (transverse magnetic) incident THz waves are illustrated in Figs. 3(a) and 3(b), respectively. The numerical calculations of the absorption spectra are carried out by using the commercial microwave software CST Microwave Studio®. The measured results are tested via a THz time domain spectroscopy (THz-TDS) system with frequency resolution of 4.58 GHz. For TE incident THz wave, as shown in Fig. 3(a) of the red line, the peak absorbance is 99.3%, and the bandwidth with absorptivity ≥ 95% is about ~1.5 THz. The measured result, as depicted in Fig. 3(a) of the blue line, also demonstrates an over 1.5 THz absorption bandwidth (absorptivity ≥ 95%). Both the calculated and the measured spectra show good agreement, except for a slight difference in resonance frequency. This discrepancy can be considered as a result of structural difference between the fabricated sample, and the calculated model. Comparing Figs. 3(a) and 3(b), we can find that for both TE and TM incident THz waves, the absorption spectra are nearly consistent with each other for the normal incidence. It means that such an ultra-broadband THz absorber is insensitive to the polarization of the incident waves, due to the symmetrical periodical structure. Such an absorption bandwidth for both TE and TM incident THz waves is much broader than that of the case in doped-silicon photonic crystal slab (Ref. 32). From Figs. 3(a) and 3(b), we can also find that three absorption peaks at 1.265 THz, 1.72 THz, and 2.155 THz appear in the spectra. Such a broadband THz absorber is caused closely combined with these three peaks. The physical mechanism of these three peaks can be explained qualitatively as follows: the left peak is induced by the anti-reflection effect, while the right two peaks are caused mainly by the grating diffractions. When the carrier density is *n* = 1.6 × 10^16^ cm^−3^ (with the resistivity of 0.54 Ω·cm), the quality factor of our designed periodical structure is very low, as shown in Fig. 3(a) (inset). Therefore, these three peaks can be broadened and combined into each other, resulting in an ultra-broadband THz absorber, as mentioned in Ref. 32. For *f* = 1.265 THz, the wavelength of the incident THz wave is larger than the period of the structure, so the grating can be considered as an effective medium. According to the effective medium theory, the effective index of the two-dimensional grating array can be calculated as^35^
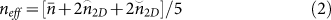
with
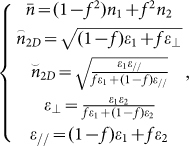
and 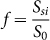 (here, *S_si_* is the surface area of the two crossed dumbbell-shaped doped-silicon strips, and *S_0_* = *p* * *p*), *n_1_* = 1 and *n_2_* is the index of doped-silicon. In order to prove the anti-reflection effect at low frequency regime (*f* = 1.265 THz), we calculate the reflection spectrum of a doped-silicon substrate coating with such effective medium (calculated from Eq. (2)) upon its upper-surface, as shown in Fig. 3(c). Obviously, it is a typical reflection spectrum, and a dip nearly at *f* = 1.265 THz with small reflection which is caused by the anti-reflection effects between the reflected waves. Here, the effective medium can be regarded as anti-reflection coating layer above the substrate, and thus, the absorption peak at *f* = 1.265 THz is caused by the anti-reflection effects. From the field distribution shown in Fig. 4(a1), it can be found that, the incident electric field is almost localized in the two crossed dumbbell-shaped doped-silicon arrays and less of incident wave is transmitted into the substrate (see Fig. 4(a2)), demonstrating that in low frequency regime, the grating arrays are equivalent to an effective anti-reflection coating, and the incident wave is mainly absorbed in this effective medium (grating layer). However, in the high-frequency regime, the reflection spectrum of Fig. 3(c) still shows a high reflection which is mismatched with the absorption characterizes in Figs. 3(a) and 3(b). That is to say, the effective medium theory can't completely apply to the high-frequency regime.

In high frequency regime, the period of the structure is larger than the wavelength in the silicon but still smaller than that in free space (*λ*/*n* < *p* < *λ*), and the periodical structure can be mainly considered as grating arrays^33^. Taking TE (electric field is parallel to x-axis) incident THz waves for example, the incident electric field will ‘see’ two crossed strip arrays with different widths along x-axis. So, two kinds of grating (horizontal stripe and vertical stripe gratings) diffractions can be realized by using these crossed dumbbell-shaped doped-silicon grating arrays. Based on two-dimensional rigorous coupled-wave analysis (2D-RCWA) method, we calculate the grating diffractions of the grating, as shown in Fig. 3(d). The two peaks at *f* = 1.72 THz and *f* = 2.155 THz are mainly caused by [±1, 0]-order, [0, ±1]-order, and [0, 0]-order grating diffractions, respectively. Furthermore, from the field distribution of Fig. 4, we can conclude that the peak at *f* = 1.72 THz is mainly due to the [±1, 0]-order field diffraction through the smaller air gap between the horizontal dumbbell-shaped doped-silicon strips (see Figs. 4(b1) and 4(b2)), and the [0, 0]-order field diffraction through the vertical dumbbell-shaped doped-silicon strips. The peak at *f* = 2.155 THz is mainly due to the [0, ±1]-order field diffractions through the bigger horizontal air gap between the vertical dumbbell-shaped doped-silicon strips, and the [0, 0]-order field diffraction through the horizontal dumbbell-shaped doped-silicon strips (see Figs. 4(c1) and 4(c2)). Comparing with Figs. 4(a2), 4(b2), and 4(c2), significant field distributions appear in the substrate (as shown in Fig. 4(b2) and 4(c2)), which means that in the high-frequency regime, the periodical arrays are considered as grating arrays and much of the incident waves is absorbed in substrate through the grating diffraction.

**Figure 5:**
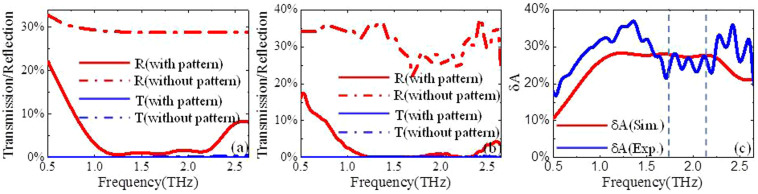
Reflection spectra and transmission spectra for the doped silicon slab with pattern (red and blue dash dot lines) and without pattern (red and blue solid lines): (a) calculated results, and (b) measured results. (c) The difference of the absorption between the doped silicon slab with pattern and the doped silicon slab without pattern: red line is the simulation result, and the blue line is the experimental result.

Although the broadband absorption in high-frequency regime is mainly caused by the grating diffractions as discussed above, it is not clear about the distinct mechanism of the absorption due to the grating diffractions. Figures 5(a) and 5(b) illustrate the calculated and the measured transmission spectra together with the reflection spectra, respectively. For the doped silicon slab with/without pattern (grating array), the transmission efficiency is nearly zero. However, the reflection efficiency is quite different from each other, and the reflection efficiency for the doped silicon slab without pattern is significantly higher than the case of the doped silicon slab with pattern. It means that the pattern on the top of the doped silicon slab plays a key role in reducing the reflection of THz wave. And, the absorption efficiencies induced by the grating diffractions are about 23% and 27% for *f* = 1.72 THz and *f* = 2.155 THz, respectively (Fig. 5(c)).

**Figure 6:**
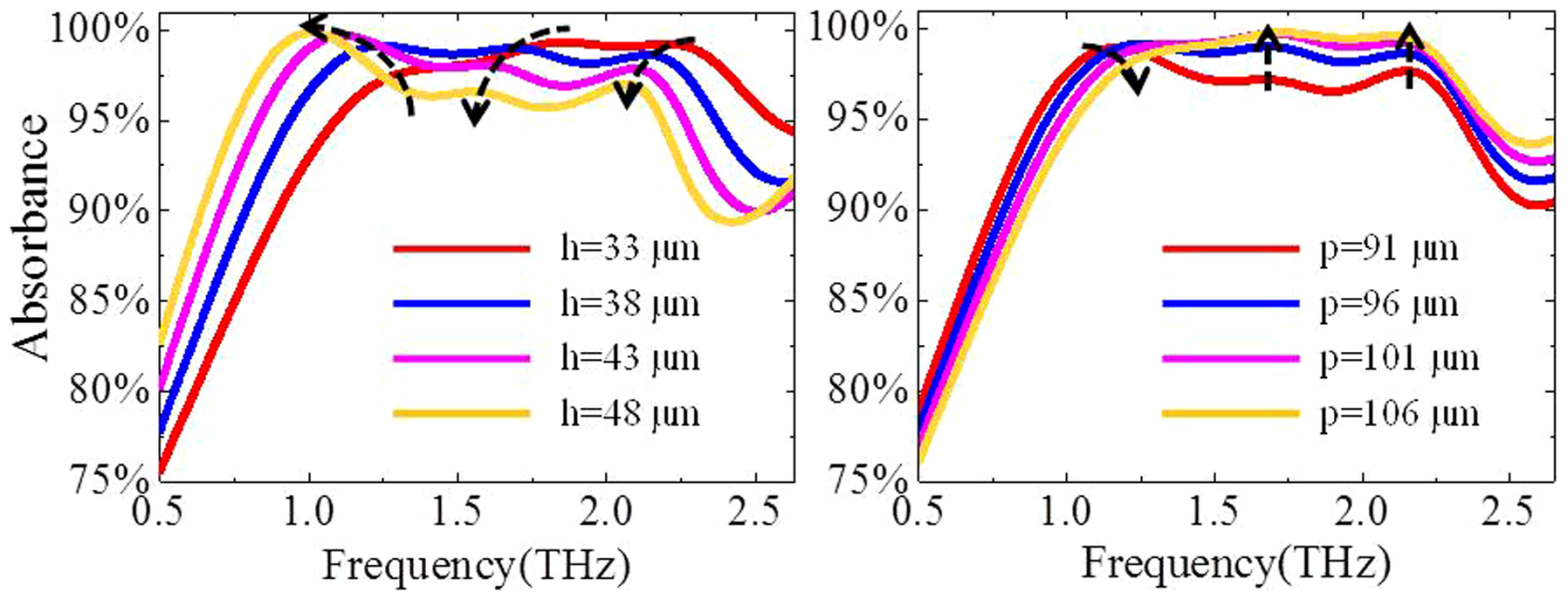
The dependences of the absorption spectra on: (a) grating depth *h*; (c) grating period *p*.

Now, we investigate the influence of the pattern (grating array) thickness and the period on the absorption spectra. Figure 6(a) shows the absorption spectra with different pattern thickness. By increasing the pattern thickness, all of the three peaks are shifted to the lower frequency (redshift). For the left peak, it is caused by the anti-reflection effects with the corresponding anti-reflection condition of *nh* = λ/4 (where *n* is the index of the doped-silicon and *h* is the thickness of the pattern). Therefore, the left peak is shifted to the low frequency with increasing of the pattern thickness (*h*). However, the other two peaks at high-frequency regime, mainly caused by the zero-order and the first-order grating diffractions, also appear the redshift effects for the increasing of the pattern thickness. This may be understood as follows: First, the grating diffraction frequencies are independent on the thickness of the pattern. Second, the redshift of the two peaks at high-frequency regime is caused by the Fabry- Pérot resonance in the pattern. That is to say, when the zero-order and the first-order diffracted waves transmit to the interface between the pattern and the substrate, a partial of diffracted THz wave is reflected upward due to the impedance mismatching. Furthermore, the reflected THz wave is mainly reflected in the interface between the pattern and the free space, leading to the Fabry- Pérot resonance in the pattern (Here, partial of the THz waves on Fabry- Pérot resonance can also be considered as separating original from the grating diffractions.). Such Fabry- Pérot resonance effect in the pattern is depended on the thickness of the pattern, and it appears the redshift effects with increasing the pattern thickness. Therefore, the two peaks in the high-frequency regime show the redshift effects. When increasing the period of the grating (the thickness and the structure size of the pattern is fixed), the left peak shows the blueshift effects while the resonance frequency of the other two peaks at high-frequency regime are nearly fixed, as shown in Fig. 6(b). The effective index of the grating is decline by increasing the period of the grating, and thus, the left peak at low frequency regime, caused by the anti-reflection effect, appears the blueshift effects (according to the anti-reflection condition of *nh* = λ/4). At high-frequency regime, the other two peaks are nearly fixed at the original resonance frequency (with the increasing of the pattern period) due to the competitive balance between the blueshift of the Fabry- Pérot resonance and the redshift of the grating diffractions. So, by adjusting the period and the thickness of the pattern, a broad-band THz absorber can be realized.

**Figure 7:**
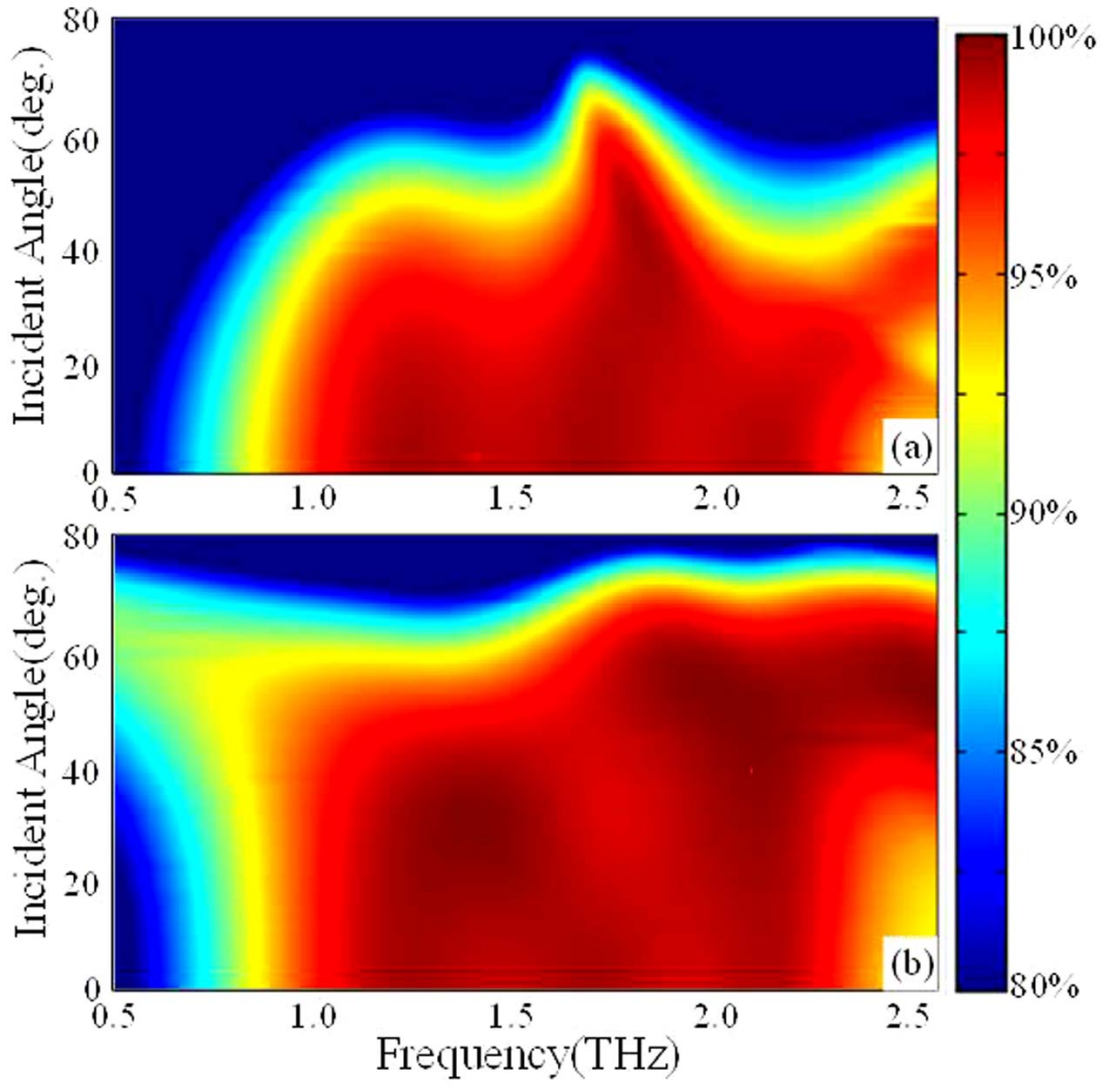
The absorption spectra with different incidence angles for (a) TE polarized THz wave, and (b) TM polarized THz wave.

For practical applications, the incident THz wave isn't always radiating normally into the THz absorbing device. Therefore, we also study the absorption characterizes of this THz absorber at various incident angles for TE and TM polarizations shown in Fig. 7. For TE polarization (Fig. 7(a)), the maximum absorption remains above 90% with bandwidth of 1.5 THz even for incident angle as large as 45°. The absorption efficiency of TM polarization is larger than 95% with bandwidth of 1.5 THz with incident angle up to 45°. All of these results verify that such a THz absorber is insensitive to incident directions (with incident angle ≤ 45°).

In summary, a novel broadband THz absorber has been proposed and experimentally demonstrated. In such a device, THz waves could be efficiently absorbed over 95% with bandwidth of 1.5 THz by etching two 90 degree crossed dumbbell-shaped grating on boron-doped silicon, which was much broader than all of the previous reported THz absorbers. Anti-reflection effect and especially the [±1, 0]-order and [0, ±1]-order grating diffractions were applied to qualitatively analyze the performance of the designed THz absorbers. The calculated results were in good agreement with the measured ones in experiments. The designed THz wave absorber device was insensitive to the polarization states of incident waves due to the symmetry structure. Our investigation may have wide applications extended to THz filters, couplers, detectors, modulators, and switches.

## Methods

### Numerical simulation

The absorption spectra and electric-field distributions are calculated by using a commercial electromagnetic simulator of CST Studio Suite® 2012. We use frequency-domain solver to acquire transmission spectra. Unit cell boundary condition was used to simulate the THz absorber. The electric-field distributions were obtained based on the time-domain solver at certain frequencies. The complex dielectric constant of *p*-type doped-silicon was determined by virtual of Drude model. In order to analyze the absorption characteristics of the periodical array at high-frequency region, two-dimensional rigorous coupled wave analysis (2D-RCWA) was used to calculate the corresponding diffraction orders, based on a state-variables representation.

### Fabrication and Testing system

The two 90 degree crossed dumbbell-shaped grating was fabricated on a *p*-type doped-silicon wafer by using the traditional photolithography and inductively coupled plasma (ICP) etching. The resistivity of the boron-doped silicon is 0.54 Ω cm. AZP4620 image reversal photoresist layer with thickness of 1 μm was spin-coated and patterned on the silicon substrate based on the standard photolithography. Then, etching for a proper time, the two crossed dumbbell-shaped doped-silicon grating is formed on the surface of the doped silicon.

The absorption characterizes of the sampler was tested based on a THz time-domain spectroscopy (THz-TDS) system. We use fiber laser to pump and detect the THz waves. The emitter and the detector are the LTG-GaAs-based photoconductive antenna. For the transmission spectra, two off-axis parabolic mirrors are used to focus the terahertz waves on the sample, and the other two off-axis parabolic mirrors collect and focus the transmit THz wave on the detector. For the reflection spectra, a 50/50 terahertz beam splitter is added into the system to gather the reflected wave from the sample. The absorption spectra are obtained based on *A* = 1 − *T* − *R* (*T* is the absorbance, while *R* is reflectance).
